# A Real Chance for the Transatlantic Partnership on Climate Policy

**DOI:** 10.1007/s10272-021-0946-0

**Published:** 2021-01-26

**Authors:** Claudia Kemfert

**Affiliations:** Depart. Energy, Traffic and Environment, German Institute for Economic Research DIW Berlin, Mohrenstraße 58, 10117 Berlin, Germany

## Abstract

The new transatlantic partnership can be the cornerstone of this change: real climate protection without false truths and hidden smoke bombs, but a shift to a full supply of renewable energies.

The future is back in the US. "We are back" indeed represents the return of the US to the world stage, and this applies not only, but most importantly, to climate. The US re-entry into the Paris climate agreement is a new page in climate policy. It is not only important symbolically. Global emissions must fall quickly, as we are running out of time. The US is the world's main greenhouse gas emitter. With the change of administrations, the world can breathe a sigh of relief. President Biden's plans are ambitious and promising and, if realised, could bring about real change. It is high time: America's reputation as a global climate protector is abysmal. For the second time, the US has helped to negotiate a climate agreement, only to withdraw from it: first from the Kyoto Protocol in 2001, then from the Paris Agreement in 2020. From now on, it can only get better. It must get better.

Above all, outgoing President Donald Trump represents the past: he wanted to save coal, build oil pipelines and cancel the climate treaty. His energy policy, a throwback to ways of 30 years ago, has largely denied the future and ignored science. But his plan did not quite work: Despite the Trump administration's policies, emissions are down as fossil natural gas has replaced coal. That is not a true energy transition to full renewables, but it is still better than nothing. Biden, on the other hand, now stands for the future: He wants to strengthen renewables, support the climate treaty and make the energy world fit for the future where digitalisation and decentralisation are important. The stakes are thus high, very high.

For one thing, the race for technologies has already begun. Coal technology, like nuclear technology, is a thing of the past. Just as we no longer use coal-fired heating systems to keep our homes warm, we will no longer need coal-fired power plants to generate electricity in the future. The old energy world does not fit into the new energy world transition. In fact, the old energy systems were based on centralised and inflexible coal and nuclear power plants that cannot offer the newly demanded flexibility of the power system. Moreover, coal-fired power plants produce greenhouse gases, and coal mining causes enormous environmental and health damage, especially in the US. Nuclear energy comes at a considerable cost as well, not only in the construction and dismantling of the plants, but especially in the disposal of the nuclear waste that has to be stored over thousands of years. Even in the US, there is no final repository for the nuclear waste. The new energy world is more decentralised, small-scale, networked and intelligent, and it is based on the intelligent networking of volatile renewable energies, storage and energy saving (Jacobsen, 2020).

**Claudia Kemfert**, German Institute for Economic Research (DIW Berlin); Leuphana University Lüneburg, Germany.

## The world is at a turning point

While we stare down the face of irreversible climate change, we can see that climate protection is finally coming to the fore. In the midst of the health crisis, we are being forced to act quickly to respond to the next imminent threat: the climate crisis. The response is coming hard and fast: heads of state and governments of the European Union are working to reduce greenhouse gas emissions by 55% below the 1990 level by 2030 (Frangoul,

2020). President Biden wants to arrange for the United States to rejoin the Paris Accord on Wednesday, 20 January, the first day of his new administration. The agreement provides that the US will reduce its carbon emissions by 25% below 2005 levels by 2025. At the same time, Chinese President Xi Jinping announced at a virtual climate summit that China would reduce its carbon emissions by 65% below 2005 levels by 2030, with renewable energy sources accounting for 25% of energy consumption by then.

Will this be enough to avert the climate crisis? Unfortunately, the answer is no. Although it is welcome news that action is now being taken and that climate protection has finally been placed at the top of the political agenda where it belongs, neither the level of ambition nor the level of implementation is sufficient to achieve the Paris climate resolutions. Furthermore, the Paris climate resolutions themselves are not ambitious enough to limit global warming to 1.5 and well below two degrees Celsius. This would require an 80% reduction in emissions by 2030 and a complete refusal of fossil fuels.

What our planet needs is a rapid transition to a 100% clean, renewable energy supply and to the storage of energy for everything, including non-energy emissions. This transition includes the electrification of almost everything – vehicles, heating and cooking in buildings, industrial processes – and the full supply of electricity. Because of the efficiency of electricity compared to fossil and nuclear energy, such electrification can reduce primary energy demand by more than 50%, but the demand for electricity will increase significantly. Electricity is the new oil. Clean, renewable energy sources include onshore and offshore wind energy, solar photovoltaics on rooftops and in power plants, concentrated solar energy, solar thermal energy for heat generation, geothermal electricity and heat, existing hydropower, tidal power and wave power. These types of electricity and heat are all provided by wind, hydro and solar energy sources. Storage includes electricity, heat, cooling and environmentally friendly, sustainable hydrogen storage. Plans have been developed for almost every country in the world to move to 100% renewable energy and storage at low cost.

The world's efforts to avert a climate crisis have not come quickly enough. As we are at least 25 years behind, we must act now. A full supply of renewable energies can be implemented quickly. During the 15 years required for the planning and construction of expensive nuclear power plants or power plants with $CO_2$ capture and storage, we could create a full supply of renewable energies instead (Child et al., 2019).

## The US must present a more ambitious climate plan

The new energy world is characterised by more decentralisation, flexibility and intelligence. Above all, it is more democratic as everyone participates in the energy transition by producing energy through solar plants or their own combined heat and power plants, providing battery storage via the electric car by means of a 'blockchain', thereby shaping the energy market in a decentralised manner themselves. California is leading the way: it builds the world's best electric cars, introduces battery storage, and in the future it also wants to offer solar tiles for the roof of the house. This is how energy transition works democratically. The USA has a choice between the new and the old, between the past and the future and between totalitarianism and narrow-mindedness or the future, intelligence, democracy and participation.

The new US envoy for climate, John Kerry, has a special role to play here. He is the right man for this major task. The Democratic US Senator from Massachusetts travelled with former Vice President Al Gore to the UN Earth Summit in Rio in 1992. In 2009, he failed to push through a $CO_2$ pricing bill in the United States. Nevertheless, he is ambitious and advocates for real climate protection. While the new administration must first address the escalating coronavirus crisis in the US and stabilise the economy, the degree of how 'green' the aid packages will be remains to be seen. Outgoing President Trump has systematically hollowed out the Environmental Protection Agency and the US State Department, responsible for climate policy, from within. This must be reversed as soon as possible.

At the climate conference in Paris, Kerry spent a week working hard in front of and behind the scenes. As Trump led the US out of the Paris Agreement, Kerry founded World War Zero in early 2019, with politicians, military leaders and actors speaking out publicly on climate policy. Kerry also chose the martial rhetoric of tackling the climate problem "like the moon landing or World War II" when he was introduced as climate envoy.

The US must quickly present a new and more ambitious climate plan. To begin with, the US could settle its bills and pay the $2 billion it still owes to the Green Climate Fund, for example. Development groups and poor countries expect that the US will not only meet its financial obligations, but hopefully also assist with the offsetting of climate damage in poor countries or cancelling debt. This is further enabled by the 5 January election results in Georgia and the resulting Democratic majority in the Senate. The goals of the Biden administration are big. With investments of $2 trillion, it wants to promote green energies, renovate six million buildings and massively expand public transportation in cities. The administration seeks to generate US electricity without $CO_2$ by 2035 and, like the EU, has set a course to be climate-neutral by 2050 (Hainsch et al., 2020).

The more ambitious the US is in climate protection at home, the more credible it is internationally. Together with China's promise of carbon neutrality by 2060 and the plans of the EU, Japan and South Korea to be carbon neutral by 2050, we are approaching a tipping point that puts the 1.5 degree Celsius target from the Paris Agreement within reach.

This is precisely why it is so important to set political decisions and framework conditions in such a way that the goals of a full supply of renewable energies can be achieved. At home, the US should do everything it can to expand renewable energy as quickly as possible. But this also means saying goodbye to the lobbyists of the past and letting the lobbyists of the future take the helm, at least putting them at the decision-making table. In Europe, too, valuable years have been lost during which coal was ramped up rather than cut back, and the sustainable transformation of transport has been delayed.

## EU-US cooperation to achieve climate goals

EU members are also making changes. Germany, for example, has decided to phase out nuclear energy by 2022 and will probably phase out coal by 2038 – most likely earlier. A full supply of renewable energies can be achieved by then if the roadmap is set today (Hainsch et al., 2020).

The challenge for many at present is that a business model that sustains the status quo under the guise of 'climate neutrality' is taking shape. Two recent examples from Europe illustrate the absurdity of current policy related to natural gas.

- Europe is in the process of constructing the Nord Stream 2 natural gas pipeline from Russia to Germany (Holz and Kemfert, 2020). This contradicts all mutual objectives and is economically and ecologically nonsensical; however, it was agreed upon as a concession to special interest groups within the framework of 'climate neutrality'.

- Recently, EU policymakers have been raving about blue hydrogen, in which the $CO_2$ from hydrogen production using natural gas is captured and stored. Such technologies are often promised by companies in the oil and gas industry as "the miracle weapon for achieving climate neutrality", for which they request generous state subsidies.

World leaders need to stop letting fossil fuel and nuclear business models continue under the guise of 'climate neutrality' and other monikers. There are only advantages to a rapid transition to real clean, renewable energy and storage (Ram et al., 2017). Germany should build alliances for a full supply of renewable energies and pursue this path together throughout Europe and with the US.

With the EU Green Deal, Europe is setting the course for real climate protection and the reduction of greenhouse gases. Under the incoming Biden administration, the US is willing to do more for real climate protection, not only nationally but also internationally. The opportunity to enact real change is better than it has been in a long time. Now is finally time to make it a reality.

The new transatlantic partnership can be the cornerstone of this change: real climate protection without false truths and hidden smoke bombs, but a shift to a full supply of renewable energies (Oei et al., 2020). The EU must work together with the USA to ensure that climate protection is a joint priority using the full power of both parties. The result will be enormous economic opportunities for both sides. Instead of sanctions and threats, Europe and the USA jointly need to focus on partnership and cooperation. The policies of the Trump administration were a shock to the system, and perhaps a necessary one to awaken and motivate climate activists and policymakers alike. We have seen how far we can fall and we never want to go back to that place again. We have been reminded of the importance of mutual respect and cooperation and its absolute necessity to achieving common goals.
